# Diverse functions for acyltransferase-3 proteins in the modification of bacterial cell surfaces

**DOI:** 10.1099/mic.0.001146

**Published:** 2022-03-07

**Authors:** Caroline Pearson, Sarah Tindall, Jennifer R. Potts, Gavin H. Thomas, Marjan W. van der Woude

**Affiliations:** ^1^​ Department of Biology, University of York, Heslington, UK; ^2^​ York Biomedical Institute, University of York, Heslington, UK; ^3^​ Hull York Medical School, Heslington, UK

**Keywords:** membrane-bound acyltransferase, *O*-acetylation, *O*-succinylation, acetyl-CoA, lipopolysaccharide, capsule, exopolysaccharide, mycolic acid, nodulation, antibiotics

## Abstract

The acylation of sugars, most commonly via acetylation, is a widely used mechanism in bacteria that uses a simple chemical modification to confer useful traits. For structures like lipopolysaccharide, capsule and peptidoglycan, that function outside of the cytoplasm, their acylation during export or post-synthesis requires transport of an activated acyl group across the membrane. In bacteria this function is most commonly linked to a family of integral membrane proteins – acyltransferase-3 (AT3). Numerous studies examining production of diverse extracytoplasmic sugar-containing structures have identified roles for these proteins in *O*-acylation. Many of the phenotypes conferred by the action of AT3 proteins influence host colonisation and environmental survival, as well as controlling the properties of biotechnologically important polysaccharides and the modification of antibiotics and antitumour drugs by Actinobacteria. Herein we present the first systematic review, to our knowledge, of the functions of bacterial AT3 proteins, revealing an important protein family involved in a plethora of systems of importance to bacterial function that is still relatively poorly understood at the mechanistic level. By defining and comparing this set of functions we draw out common themes in the structure and mechanism of this fascinating family of membrane-bound enzymes, which, due to their role in host colonisation in many pathogens, could offer novel targets for the development of antimicrobials.

## Introduction

Within biological systems, many small molecules are chemically modified during or after synthesis with additional functional groups to alter their chemical properties. For example, a well-known modification to the antibiotic chloramphenicol is catalysed by chloramphenicol acetyltransferase (CAT), which is used by bacteria to confer resistance to this antibiotic by modifying it such that it can no longer bind efficiently to its target site [1]. The soluble CAT enzyme is cytoplasmic and uses the abundant central metabolite acetyl-coenzyme A (CoA) as a source of activated acetyl groups for the catalytic addition onto the antibiotic.

Bacteria have many soluble cytoplasmic acyltransferases that function in normal metabolism, but, in addition, it is known that many cell-surface structures that contains sugars, such as the lipopolysaccharides, mycolic acids, peptidoglycans, exopolysaccharides and glycosylated proteins, can also undergo acylation. These processes have usually been identified as they have some relation to altering bacterial virulence. For example roles for *O*-acylation of glycans by acetyl-, propionyl-, succinyl- and many other acyl substituents are known to play roles in diverse processes such as antigenic variation [2–4], osmoregulation [5], virulence [6] and cell division [7]. Furthermore, they are critical for modification of secreted or extracellular polysaccharides that are involved in many bacterial interactions with their environment, from niche competition [8] to initiation of symbiosis [9] and biosynthesis of antibiotics and antitumour drugs [10].

All the complex surface structures mentioned above require at least partial biosynthesis in the cytoplasm, then movement of components across the inner membrane and then final assembly either during export or post-export in an extra-cytoplasmic location. Chemical modification of these building blocks by acylation also often occurs in a similar extra-cytoplasmic location, which poses a problem for the cell in having to export activated acyl groups to be attached to diverse chemical structures. The solution appears to be the evolution of membrane-bound acyltranferase enzymes, which can not only use a source of activated acyl groups, likely acyl-CoAs, but also move the acyl group across the membrane and attach it to the sugar in an extracytoplasmic location.

Looking at the proteins responsible for these modifications, we find two known families that have been characterised to different levels of understanding. The first, which is the most abundant and involved in the most diverse processes in bacteria are proteins containing the acyltransferase-3 (AT3) domain (IPR002656, PF01757), referred to here as AT3, and which is the focus of this review. While the diversity of modified cell structures that AT3 proteins generate will become apparent in the following pages, relatively little is known about their mechanism of action [11]. The AT3 domain contains ten membrane spanning helices and their molecular function is classified by the InterPro database as having ‘transferase activity, transferring acyl groups other than amino-acyl groups’ [12]. Although present in all domains of life, most research on AT3 proteins has been conducted in bacterial species and there are limited examples of functionally characterised AT3 domain-containing proteins in eukaryotic systems. Current examples of the latter include proteins in *Caenorhabditis elegans* and *Drosophila* involved in development and drug responses [13, 14]. However, these studies are limited to knockout and complementation of the AT3-encoding genes and their exact functions, including the targets of their acyltransferase activity, are currently unknown.

The second known family of membrane-bound acyltransferases is also found in all domains of life and are known as the membrane bound *O*-acyl transferase (MBOAT) proteins (IPR004299, PF03062). The MBOAT superfamily was first defined by Hofmann [15] after finding sequence similarities between a eukaryotic cholesterol acyltransferase (ACAT1) and a protein acetyltransferase (Porcupine) involved in embryogenesis. Members of this protein family share the common features of multiple transmembrane helices containing a functionally important histidine residue and in some cases a critical asparagine residue [16, 17]. These proteins have been studied in animals, plants, fungi and bacteria and display a diverse range of acyltransferase activities important in many biological processes. They have defined roles in acylation of secreted signalling proteins [18], lysophospholipids [19] and glycans [20]. The use of MBOAT proteins in bacteria currently appears more limited than that of AT3 proteins. Some of the best studied are the alginate *O*-acetyltranferase AlgI involved in alginate biosynthesis in species of the genus *Pseudomonas* [21, 22], the PatAB system for peptidoglycan *O*-acetylation in *Neisseria meningitidis* [23] and the d-alanyl transfer protein DltB, involved in teichoic acid biosynthesis in Gram-positive bacteria, although here the MBOAT protein functions to transfer the d-alanyl group rather than an acyl group [24]. The first crystal structure of an MBOAT protein has been recently elucidated [25], revealing a totally new fold for a membrane protein and shedding some light on the mechanism of these enzymes. In this review we will only mention them in the context of processes where both AT3 and MBOAT proteins have evolved identical functions, which currently appears uniquely in the case of peptidoglycan *O*-acetylation [7].

To understand the function and diversity of AT3 proteins, we first discuss their overall architecture, and then present the first compilation, to our knowledge, of bacterial systems that are known to use AT3 proteins as part of a cellular processes. It is hoped that this compilation will facilitate cross-system testing of hypotheses regarding mechanisms of action and inform studies to maximize their potential applications.

## Architecture of AT3 proteins

**Fig. 1.**
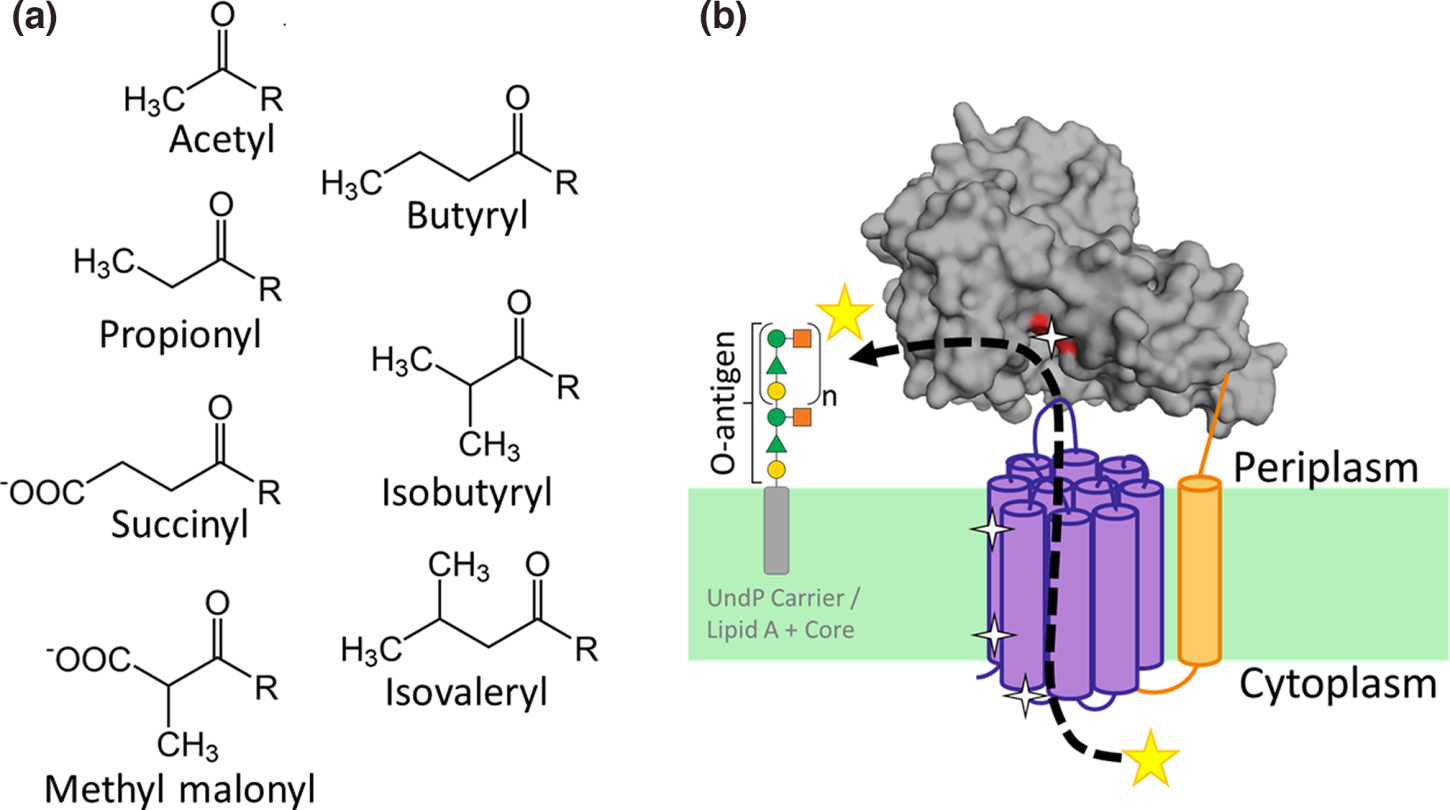
(**a**) Acyl modifications known to be added to sugars by AT3 proteins mentioned in this review. Note that only some modifications alter the charge of the sugar. (**b**) Chematic model of OafB adapted from [11]. The known structure of the OafB SGNH domain (PDB:6SE1) (grey) is shown oriented via TMH11 and the periplasmic linking region (orange) and the AT3 (purple). Positions of residues known to be essential for function are indicated by white stars. Yellow stars represent the acyl-group being transferred to the O-antigen on the outer leaflet of the inner membrane.

AT3 domain-containing proteins were first proposed to be a family of integral membrane proteins responsible for trans-acylation modifications by Slauch *et al.,* [2] in 1996. This paper identified several proteins involved in acylation of sugar moieties that were similar to OafA, the protein responsible for acetylation of the *Salmonella enterica* subsp. *enterica* serovar Typhimurium (STM) O-antigen. They were termed integral membrane trans-acylase family proteins, later to be defined by the InterPro database as acyltransferase_3 (acyl_transf_3 or AT3) family proteins. An assessment of the Interpro or Pfam entry for AT3 domains (IPR002656/PF01757) reveals that they occur most frequently as stand-alone domains with ten predicted transmembrane helices (TMH). The most common type of domain fusion is a C-terminal fusion of the AT3-domain to a serine, glycine, asparagine and histidine (SGNH) domain, which we refer to as AT3–SGNH proteins, and these fused proteins have an additional 11th TMH to provide the correct topology to locate the SGNH domain in an extracytoplasmic location (Fig. 1).

SGNH domains have a slightly confusing history and were first identified as a subgroup of the glycine, aspartate, serine and leucine (GDSL) hydrolase family, with their transferase activity being characterised at a later date. The GDSL family, first described by Upton and Buckley [26], is characterised by five catalytic blocks which between them contain residues that form a Ser–His–Asp/Glu catalytic triad in the enzyme. The SGNH-hydrolase family emerged as a subgroup of this family when the acetyl esterase proteins rhamnogalacturonan acetylesterase (RGAE) and serine esterase from *Streptomyces scabies* (SsEst) were classified as members of a novel family of hydrolases due to conservation of catalytic residues within only three of the five GDSL catalytic blocks (I, III, V) [27]. Subsequently, the crystal structure of RGAE was solved [28], which enabled identification of other related protein structures with a similar α/β/α hydrolase fold, but relatively low sequence identity. Alignments with this wider list of acyl esterase proteins revealed conserved residues corresponding to block II of the GDSL catalytic blocks [28]. Finally the SGNH-hydrolase family name was coined in 2000 due to the finding that one single residue in each of the four catalytic blocks identified was conserved and catalytically important (S-Block I, G-Block-II, N-Block III, H-Block V) [28].

Although having known *O*-acetylesterase activity, the SGNH domain is essential for the acyltransferase activity of AT3–SGNH fused carbohydrate acyltransferases [4, 11, 29, 30] (Fig. 1). Furthermore, periplasmic sequence unique to the fused AT3–SGNH proteins seems to be involved in restricting acceptor substrate specificity [11]. Most recent publications on AT3 domain containing acetyltransferases describe a proposed general predicted mechanism that an acetyl group is transported from a donor in the cytoplasm across the inner membrane by the AT3 domain where it is subsequently transferred to the acceptor carbohydrate on the extracytoplasmic side [11, 31, 32] (Fig. 1). For AT3-only proteins it is thought that the AT3 domain alone is responsible for the whole transport and transfer reaction, however for AT3–SGNH fused proteins it is thought that the SGNH domain is responsible for the final transferase reaction. This raises many questions: first, how are AT3-only proteins able to function without the fused SGNH domain? Or is a partner protein, SGNH or otherwise, required? How do AT3-only proteins differ in their structure to enable transport and transfer while AT3 domains in AT3–SGNH proteins are only able to perform the transport reaction? Finally, does the requirement for the SGNH domain relate to the nature of saccharide acceptor and how it is presented to the enzyme?

The results of mutational analyses of the single domain AT3 protein OacA encoded by *Shigella* spp phage [32, 33], revealed that there are specific residues which are essential for function; some of these are part of a shared motif as per Interpro logo for the entire AT3 family. These motifs are, for example, the RXXR motif at the first cytoplasmic loop [32, 33] and the R/K-X10-H motif in TM1 for OafA/OafB [11] (Fig. 1). While this review is about the biological functions of the AT3 proteins and there are no known structures, there are structural models based on topological predictions mapping conserved residues from work on three different systems mentioned in the review, namely Oac, OafA and OatA [11, 30, 33]. These studies were based on *in situ* functional assays. To understand further the role of these essential residues, development of *in vitro* assays with purified protein and structural information will be required to clarify the enzymatic action and roles of specific residues, and to decipher the mechanism of action of AT3 proteins.

**Fig. 2.**
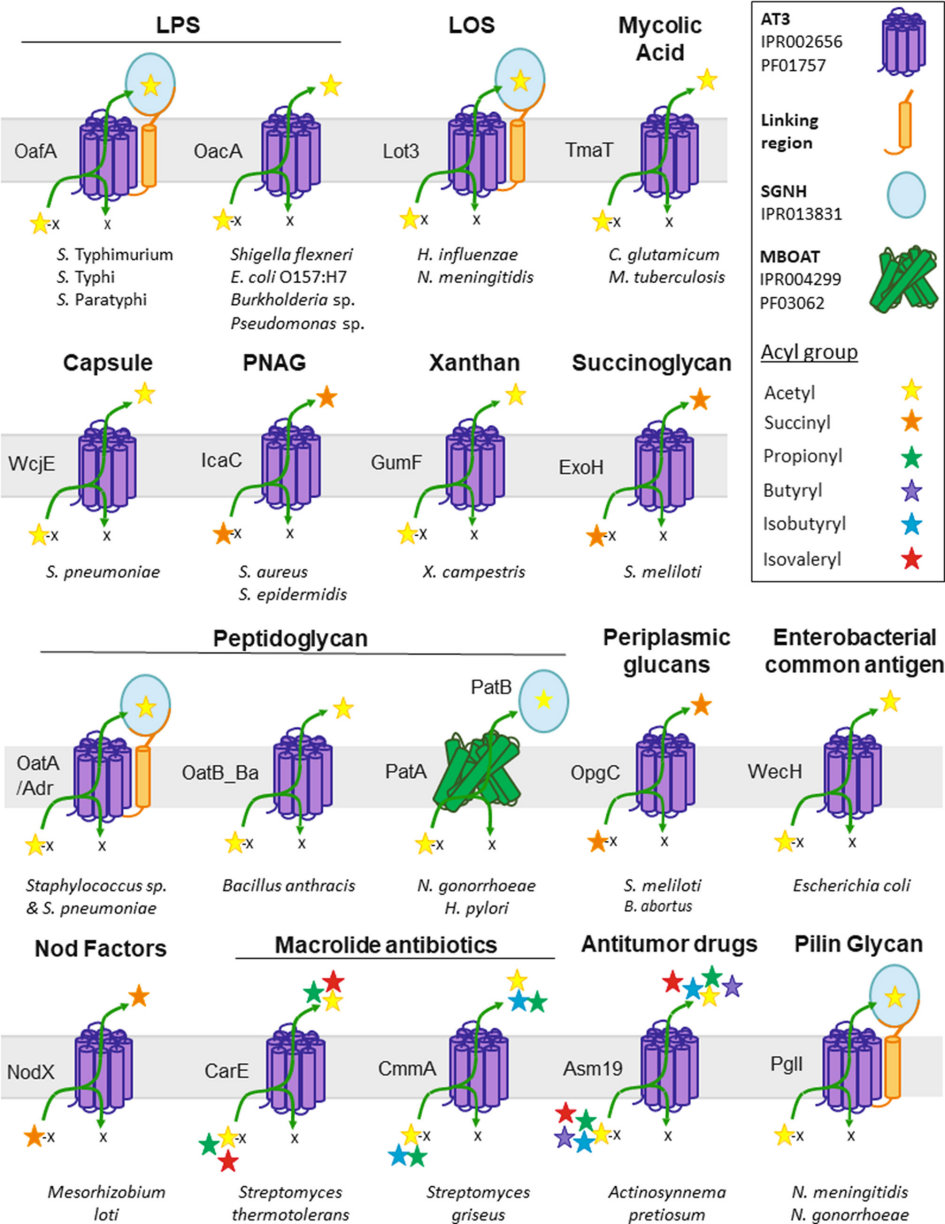
Schematic diagram representing the functional diversity of different targets for *O*-acylation by AT3 proteins, their domain structures and acyl donors that have been experimentally demonstrated to be used by each enzyme.

To understand the importance of AT3 proteins in bacterial function, we have collated information of all functionally studied phenotypes where AT3 proteins have been implicated. These are presented and discussed here for the first time, to our knowledge (Fig. 2).

## Key carbohydrate targets of bacterial AT3 proteins

### Lipopolysaccharide O-antigen

Lipopolysaccharide (LPS) is the major lipid-bound carbohydrate component of the outer leaflet of the outer membrane of Gram-negative bacteria. It is considered an essential component of the cell envelope of many Gram-negative bacteria as it plays roles in the correct assembly and function of outer membrane proteins and prevents the passage of toxic hydrophobic molecules into the cell [34, 35]. LPS is composed of a membrane anchored lipid A, a core oligosaccharide portion, and a distal O-antigen [36]. Its lipid A portion is a key activator of innate immunity [37], while the core oligosaccharides connect lipid A to the O-antigen and are generally well conserved in composition, particularly in the inner core region [37]. The O-antigen is the most distal and variable component of LPS and it plays a pivotal role in bacterial interactions with the external environment. The O-antigen is composed of repeating oligosaccharide units of three to six monosaccharides, which can be linear or branched [38]. As the most exposed molecules to host defences, O-antigens can often represent the dominant antigen recognised by the host immune system, and can also be the target of bacteriophage interactions required to initiate infection [39]. For this reason, bacteria possess mechanisms to alter their O-antigen composition and structure. A diverse range of modifications can be performed, which include adding extra moieties, such as glucosyl and acetyl groups [2, 40].

In STM there are currently two defined O-antigen acetyltransferases OafA and OafB, which both possess AT3–SGNH fused domain architecture (Fig. 2). OafA was discovered by Slauch *et al.,* [2] to be responsible for acetylating the 2-hydroxyl group on the d-abequose moiety of the STM LPS O-antigen. This modification alters the immunological properties of *Salmonella* LPS and results in seroconversion to O:5 [41] and is also required for production of protective antibodies against invasive nontyphoidal Salmonella disease [42]. In addition to d-abequose acetylation, multiple *Salmonella* serovars contain an l-rhamnose moiety on the O-antigen which can be *O*-acetylated [43]. The protein responsible for l-rhamnose acetylation in STM was first discovered through analysis of a family II g*trC* gene encoded on the BTP1 prophage associated with STM D23580, a major cause of invasive multi drug resistant iNTS in sub-Saharan Africa [40, 44]. It has now been renamed OafB by Pearson *et al.* [11] as, rather than being responsible for the O-antigen glycosylation associated with other Gtr operons, this protein is responsible for acetylation of O-antigen rhamnose at the 2- and 3- hydroxyl groups [4, 45]. O-antigen rhamnose acetylation has been shown to protect *Salmonella* from lysis by the BTP1 bacteriophage and is thought to act through steric hindrance of the BTP1 tailspike protein, which has rhamnosidase activity and would usually cleave the O-antigen, allowing access to the cell surface [4]. *Salmonella enterica* subspecies *enterica* serovar Paratyphi A (SPA) also possesses acetylation at rhamnose 2- and 3- hydroxyl groups [46] and a homologue of OafB has been identified through sequence analysis, which is proposed to be responsible for this modification in *Salmonella enterica* ser. Typhi [45]. In S. *enterica* ser. Typhi this rhamnose acetylation was shown to be controlled by epigenetic phase variation, allowing the phenotype to be switched ‘on’ and ‘off’ within a bacterial population, and it has been found to be an immunodominant modification [45]. Phase variation signature sequences are found upstream of *oafB* sequences in multiple serovars [40, 47]. Thus, O-antigen rhamnose acetylation is involved in modulation of bacteriophage interactions and its phase variable expression could promote persistence in the host.

In a similar process, the WbaK protein (orf17.4) has also been implicated in the acetylation of the C6 of the galactose residue in the *S. enterica* serogroup E1 O-antigen [48]. However, unlike OafA and OafB, this protein has AT3-only domain architecture so does not contain a fused SGNH domain (Fig. 2). An additional contrast is that whilst the *oafA* and *oafB* genes reside at loci distinct from the O-antigen biosynthesis gene cluster in the *Salmonella* genome, the *wbaK* gene is closely associated with O-antigen synthesis genes and is predicted to have been inserted into the end of the gene cluster by homologous recombination [48]. Interestingly the ε15 bacteriophage can convert E1 *S. enterica* to group E2, which is at least partly due to the inhibition of the O-antigen acetylation modification, and, as such the phage has been proposed to encode two inhibitors of AT3 function that have not been investigated to date [49].

In addition to its significance in *Salmonella,* O-antigen acetylation is an integral feature of serotype conversion in the Gram-negative bacterial pathogen *Shigella flexneri.* Oac, one of the most well-studied ‘AT3-only’ members of the AT3 family, has been shown to catalyse 2-O-acetylation of l-rhamnose (RhaI) of the tetrasaccharide repeat (RhaIII–RhaII–RhaI–GlcNAc) of the *Shigella* O-antigen shared by all serotypes of *S. flexneri* except for serotype 6 (Fig. 2). It was independently identified by both Verma *et al.* [3] and Clark *et al.,* [50] through investigating the ability of bacteriophage *SF6* to antigenically convert *S. flexneri* serotypes through acetylation of the LPS O-antigen. O-antigen acetylation caused by the *SF6* phage causes immunity to superinfection by further *SF6* phage as the modification sterically hinders its endorhamnosidase activity. A second *oac* gene was identified in *S. flexneri* strain 1b by Sun *et al.,* [51], which was named *oac1b* and thought to be carried by a different phage that is present in only 1b strains. Two further *S. flexneri* O-antigen acetylation modifications were identified by Perepelov *et al.,* [52], including 3/4-*O*-acetylation of RhaIII and 6-*O*-acetylation of GlcNAc. This finding resulted in the identification of further Oac proteins in *S. flexneri* responsible for these modifications, prompting the original Oac protein to be re-named to OacA. OacB was found to be responsible for the 3/4-*O*-acetylation of RhaII in serotypes 1 a, 1b, 2 a, 5 a, and Y but not serotype 6 [53], whereas, OacC is responsible for the 3/4-*O*-acetylation of RhaII specifically in serotype 6 [54]. The *oacD* gene was found to be present on the serotype converting bacteriophage *SfII* genome and identified as the *oac* homologue responsible for the known 6-*O*-acetylation of GlcNAc by Sun *et al.* [55]. A homologue of *oac* has been identified in an *Escherichia coli* O157:H7 bacteriophage, ΦV10, which also acts in serotype conversion by *O*-acetylation of the O-antigen at an unidentified sugar position [56] and homologues more recently have been found in the *Enterobacter* bacteriophage Tyrion [57].

*Lag1* was identified by Zou *et al.,* [58] as the gene responsible for altering the reactivity of *Legionella pneumophila* LPS to serogroup 1 LPS monoclonal antibodies. The results of NMR analysis revealed that *lag1* mutants failed to acetylate position 8 of the legionaminic acid moiety in the LPS O-antigen, which has a similar structure to sialic acid [58, 59]. Like the Oac proteins, Lag1 displays AT3-only domain architecture. The acetylation modification of *Legionella* LPS by Lag1 has been associated with virulence of serogroup 1 LPS strains [60], highlighting the clinical importance of this modification. Zähringer *et al.,* [61] determined that 8-*O*-acetylation of legionaminic acid causes increased hydrophobicity of *Legionella* cell surface and suggested that this may enable stable aerosol production, which is a major source of transmission of Leigonnaires’ disease. Some functional analysis of Lag1 has been conducted by Luck *et al.,* [62]; they showed that a serine to leucine amino acid change in the highly conserved motif V–X–X–F–F–X–(I/V/L)–S–G–(F/W/Y), which is shared among many AT3 proteins from both Gram-positive and Gram-negative bacteria, was responsible for loss of *O*-acetyltransferase activity of Lag1. The mechanistic role of this single amino acid is not yet characterised and there is no *in vitro* assay for the acetylation modification. AT3 proteins have also been identified in the ß-proteobacteria *Burkholderia pseudomallei* and *Burkholderia thailandensis,* which are responsible for 4-*O*-acetylation of 6-deoxy-α-l-talopyranose of the LPS O-antigen [63], and a second gene, denoted *wbiA*, was found to be responsible for acetylation of the same O-antigen sugar at the C2 position [64].

The role of O-antigen modifications in plant–microbe interactions has also recently been discovered in the plant-root associated γ-proteobacterium *Pseudomonas protegens* CHA0, which contains an O-antigen cluster with an AT3–SGNH encoding gene present [65]. Evolution of improved rhizosphere competence by this bacterium appears to select for disruption of this gene, indicating that cell surface decoration plays some role in root-colonisation [66].

Considering one of our questions about the occurrence of AT3 versus AT3–SGNH proteins, these O-antigen systems provide the clearest examples of highly similar acceptor molecules, i.e. the lipid anchored O-antigen repeat unit, where one bacterium uses an AT3–SGNH and another appears to use a stand-alone AT3 for the same function, viz. OafA and OafB as AT3-SGNH proteins in *Salmonella* ser. Typhimurium compared to AT3 only proteins in *S. enterica* serogroup E1 and *Shigella *, among other bacteria. While there is a possibility of a ‘missing’ SGNH or similar domain in the stand-alone systems, this higher occurrence of AT3 only versus AT3–SGNH proteins, seen in many of the other systems mentioned in this review, strongly indicates that the AT3 can function alone and the fusion is conferring some additional, as yet unknown, benefit to the process.

### Lipooligosaccharide (LOS)

Some organisms which reside on mucosal surfaces, such as *Neisseria gonorrhoeae* and *Haemophillus influenzae*, possess a form of LPS that does not contain the distal repeating O-antigen and instead has a single non-repeating oligosaccharide chain [67, 68]. This alternative glycolipid is termed lipooligosaccharide (LOS). Although it does not possess the highly variable O-antigen structure, LOS is still a major virulence factor in these bacteria [69, 70], and its oligosaccharide composition varies depending on expression of biosynthetic glycosyltransferase genes [71, 72].

Perhaps not surprisingly, similar AT3-mediated modifications known for LPS occur for LOS and a number have been experimentally characterised. An early identification of an AT3-encoding gene was in the *H. influenzae* Rd genome, which at the time of sequencing was of unknown function [2]. In the original Rd genome sequence, this gene had a frameshift [73], however, in other clinical strains, such as strain Eagan, it is intact and encodes a functioning AT3 proteins that *O*-acetylates heptose-III of the LOS inner core, resulting in increased serum resistance to complement-mediated killing [73]. The expression of this gene, now called *oafA* is phase variable and on/off switching of this gene has been associated with a switch between carriage and invasive isolates of non-typeable *H. influenz* ae [74, 75].

Similarly, in *Neisseria meningitidis* an AT3 protein, Lot3, is responsible for acetylation of the C3 position of GlcNac in the inner core of meningococcal LOS [76] (Fig. 2). LOS of *N. meningitidis* is an important virulence factor, which is involved in the interaction with host epithelial cells and induces pro-inflammatory responses during human infection [77, 78]. Jennings *et al.* [79] found that *O*-acetylated LOS are more immunoreactive, producing higher antibody titres than their non-acetylated counterparts.

### Mycolic acids

In addition to Gram-negative bacteria, many other bacteria contain structures beyond the cell wall that form an effective outer membrane structure that protects the cell from various external stresses. The cell envelope of the Corynebacteria contains long-chain fatty acids known as mycolic acids that are modified with sugars, including trehalose, forming trehalose corynomycolates (TMCMs). The TMCM structure is *O*-acetylated, which requires the stand-alone AT3 protein TmaT [80] (Fig. 2). Remarkably this modification is on the fatty acid moiety of the corynomycolic acid and not the sugar component, making this a unique substrate for an AT3 [80]. A strain lacking *tmaT* cannot produce the acetylated mycolic acid and has defects in final targeting of the TMCMs to the outer membrane [80, 81]. The authors were also able to make cell-free extracts and using [^14^C]-acetyl-CoA they could see direct transfer of the label onto the TMCM. It is of note that the orthologue of TmaT in *Mycobacterium tuberculosis *, Rv0228, is encoded by an essential gene, indicating the importance of this process. In this important pathogen [82] the major mycolic acid is known to be 6-mycolyl-6'-acetyltrehalose (MAT) with the acetyl group modification on the sugar [83], which is a more typical location for an AT3 protein target. The results of these studies provide multiple mechanistic insights, firstly the demonstration that acetyl-coA is the donor substrate, and secondly the potential for broader functions than those usually considered for AT3 proteins in that a fatty acid could serve as an acceptor rather than a sugar.

### Capsule and other exopolysaccharides (EPS)

In addition to LPS and teichoic acids on the outer surface, many bacteria also produce a capsule, defined as a gel-like layer of polysaccharide that can be up to 10 µm thick [84]. These capsular polysaccharides are often negatively charged and display vast structural diversity in their monosaccharide composition, branching and linkages [85]. They can be linked to the bacterial cell surface with a lipid moiety or covalently anchored to the peptidoglycan of Gram-positive bacteria [85, 86]. Capsules have diverse functions in virulence and can, for example, be antiphagocytic and prevent complement-mediated killing [87]. They can also play roles in adherence to surfaces and other cells during formation of biofilms [88], and can provide resistance to desiccation and disinfectants [89], among other biological functions. As cell surface polysaccharides they have been targeted in vaccine research and are components of conjugate vaccines against *Streptococcus pneumoniae* [90], *Neisseria meningitidis* [91], *Salmonella enterica* serovar Typhi [92] and *Haemophilus influenzae* [93].

In the Gram-positive pathogen *Streptococcus pneumoniae* the capsule is synthesised by the products of the *cps* locus, and the first link to an AT3 functioning in this context was the discovery of a new serotype, 11E, where an AT3-encoding gene, *wcjE,* within the *cps* cluster had been inactivated [94] (Fig. 2). The initial data indicated that WcjE is responsible for the *O*-acetylation of the 1-phosphoglycerol residue in serotype 11Aα capsular polysaccharide. Calix *et al.* [95], subsequently discovered that WcjE in serotype 9V is responsible for 6-*O*-acetylation of the capsular polysaccharide β-*N*-acetylmannosamine. Thus, it is possible that various *wcjE* genes across different serotypes could perform different acetyl modifications to the capsular polysaccharide, modulating interactions with its environment. Indeed, another *cps* encoded AT3 protein, WciG, was identified to specifically acetylate carbon 2 of 6-β-d-galactofuranose in the capsular polysaccharide of a serotype 35C *S. pneumoniae* isolate [96], and the WcjE homologue of this isolate specifically acetylated carbon 5 and 6 of the 3-β-galactofuranose sugar [97]. These modifications, all catalysed by stand-alone AT3 proteins, produce varied biological properties within pneumococcal serotypes, such as altered opsonophagocytic killing, biofilm formation and adhesion to nasopharyngeal cells [98].

There are other examples from important pathogens of capsules that contain *O*-acetylated sugars, where the biosynthetic mechanism enable the sugars to be acetylated in the cytoplasm, then polymerised and exported in this modified form. Notable examples include the *O*-acetylation of the polysialic acid capsule of *E. coli* K1 by NeuO [99] and the *O*-acetylation of capsular polysaccharide in the Group B *Streptococcus* by NeuD [100], which do not use AT3 proteins. Both acetylation within the cytoplasm by NeuD, and the use of an AT3 protein to transport the acetyl group across the cytoplasmic membrane (by WcjE and WciG) are found within Streptococci isolates. This raises the question of why two contrasting mechanisms are required to produce the same end result.

The second major secreted polysaccharide with an important connection to microbial virulence is the poly-*N*-acetylglucosamine (PNAG) EPS produced by biofilm-forming *Staphylococci* [101]. Strains that produce PNAG are frequently associated with infections of inserted medical devices, with the PNAG forming an important part of the biofilm matrix [102]. GlcNAc moieties of PNAG can be *O*-succinylated and this modification is suggested to be catalysed through the action of the AT3-only IcaC protein, encoded in the *icaADBC* operon [103, 104] (Fig. 2). IcaC was originally thought to be responsible for transport of PNAG through the *Staphylococcal* cell membrane during biofilm formation [105]. However, due to its homology to other AT3 proteins it has since been suggested to *O*-succinylate the GlcNAc residues of PNAG during their coupled biosynthesis and export by IcaAD [104]. As well as this AT3-mediated modification, the PNAG can also be modified by *N*-deacetylation by the action of IcaB, which together alter the physiochemical properties of PNAG to assist in adherence and biofilm formation on a range of different surfaces [103]. A role for IcaC in formation of mature biofilm has also been identified [105]. Therefore, identifying the biochemical function of IcaC, either as a PNAG transporter and/or a PNAG *O*-succinyltransferase is an outstanding problem in Staphylococcal biofilm biology.

Turning to more environmentally and biotechnologically important EPS, there are functions for AT3 proteins in the *O*-acetylation of xanthan, an EPS produced by *Xanthomonas campestris* that has a wide commercial application as a thickening agent [106]. It is composed of a cellulose-like backbone of β-(1-4)-linked glucose with side chains composed of d-mannose and d-glucuronic acid [106]. The GumF and GumG proteins, both stand-alone AT3 proteins, were identified by Katzen *et al.* [107] as the proteins responsible for this *O*-acetylation, with GumF acetylating the mannose residue closest to the glucose backbone, and GumG acetylating the terminal mannose of the side chain [107] (Fig. 2). Acetylation of xanthan has been shown to affect its viscosity and this has many industrial applications [108, 109].

Succinoglycan is an acidic calcoflour-binding EPS, which is important for effective induction of root nodule formation by *Sinorhizobium meliloti* on alfalfa plants [110]. It is composed of repeating octasaccharide subunits which have acetyl, succinyl, and pyruvyl substituents [111], indicating promiscuity of donor substrate. Succinylation and acetylation of succinoglycan have been shown to be performed by ExoH and ExoZ, respectively. ExoH was identified as a membrane protein required for the C-6 succinylation of glucose 7 of succinoglycan [112], while ExoZ was identified by Buendia *et al.* [113], to affect succinoglycan production. It was later discovered that this protein was responsible for the acetylation of C6 of glucose of *S. meliloti* succinoglycan [114]. They are both stand-alone AT3 proteins (Fig. 2). Lack of these modifications results in ineffective root nodulation [112, 115, 116].

It is worth noting that there are additional EPSs that are modified by *O*-acetylation in an extracytoplasmic location that use an MBOAT protein instead of an AT3 protein to deliver acyl groups across the membrane. These includes alginate in *Pseudomonas aeruginosa *, that uses the MBOAT protein AlgI in combination with a separate SGNH protein (AlgX) [117] and also the WssH MBOAT protein that is responsible for cellulose *O*-acetylation in *Pseudomonas fluorescens* [118]. Finally, there are additional EPSs that are known to be *O*-acetylated where the mechanism is still completely unknown and where AT3, MBOAT or some other system could be involved, for example the EPS-2 from *Lactobacillus johnsonii* FI9785 [119].

### Peptidoglycan

One of the most well-studied and biological important phenotypes conferred by an AT3 protein-mediated *O*-acylation process is the modification of peptidoglycan by *O*-acetylation. The structure of the peptidoglycan is comprised of a disaccharide repeat of *N*-acetylglucosamine (GlcNAc) and *N*-acetylmuramic acid (MurNAc), crosslinked with peptides that bridge across the MurNac molecules [120]. While the peptide stem can vary across bacteria [121], the carbohydrate composition of glycan strands is regarded as uniform and is the target of the human hydrolytic enzyme lysozyme. *Staphylococcus aureus* can *O*-acetylate carbon 6 of MurNAc in peptidoglycan [122], which confers resistance to lysozyme [123].

Bera *et al.* [6], were able to identify *oatA* as the gene responsible for this peptidoglycan acetylation in *S. aureus.* They showed that OatA, an AT3-SGNH fused protein (Fig. 2), was required for this specific modification of MurNAc. In the important human pathogens *S. pneumoniae* and *Listeria monocytogenes* the homologous proteins, Adr and OatA respectively, catalyse the same reaction and its disruption results in sensitivity to penicillin and lysozyme [124, 125], while the OatA protein in *Lactococcus lactis* modifies peptidoglycan in the same way [126]. All these proteins are AT3–SGNH fusions. Intriguingly a variation on this was discovered in *Lactobacillus plantarum* where the *O*-acetylation occurs on the GlcNAc sugar as well as the MurNAc sugar and in fact the bacterium has two AT3–SGNH proteins, named OatA and OatB, which catalyse the additions, with OatB being the enzyme which modifies the GlcNAc position [127]. In an important recent study it was determined that acetyl-CoA is the acetyl donor for this protein and that the activity of the SGNH domain is essential for the forward transferase reaction where acetyl-CoA is the donor for acetylation of a peptidoglycan fragment through the action of both the AT3 and SGNH domains together [30]. This paper also provided further mechanistic insights in providing evidence for conserved tyrosine residues in the AT3 domain being involved in the catalytic process and presented a revised topological model for OatA [30].

While OatB is a model for this process in *Staphylococci*, the Gram-negative pathogen *Neisseria gonorrhoeae* uses an MBOAT protein, PatA, to catalyse the same reaction, in combination with an unfused SGNH domain, PatB [23]. There is also evidence in *Helicobacter pylori* for a PatA protein having a similar role in peptidoglycan *O*-acetylation, which, in combination with peptidoglycan *N*-deacetylation, is important for survival of this pathogen in the host [128]. Here the *patA* gene is linked to another likely SGNH encoding gene, dubbed *ape3* [20].

A final twist on the evolutionary solution to peptidoglycan *O*-acetylation is evidenced from *Bacillus anthracis *, where peptidoglycan acetylation is an important factor controlling autolysins as well as having a role in S-layer anchoring [7, 129]. Both an AT3 protein, OatA, and a PatA-like MBOAT protein function in this pathogen and contribute to full peptidoglycan *O*-acetylation and the AT3 protein uniquely lacks the SGNH domain seen in other OatA/Adr proteins [7, 130] (Fig. 2). Again, this could provide an excellent experimental system to try and understand why a partner SGNH domain is required, or whether perhaps in this case the PatB SGNH domains can cross-talk to the OatA AT3 domain.

### Periplasmic polysaccharides

As well as being on the cell surface, various microbial glycans in Gram-negative bacteria also reside in the periplasm, including diverse periplasmic glucans and the cyclic form of the enterobacterial common antigen (ECA).

The osmoregulated periplasmic glucans (OPGs) form a group of structurally varied oligosaccharides composed solely of d‐glucose, that are thought to provide resilience of bacteria against osmotic stress [131] and can be important for virulence [132, 133]. Although only composed of d-glucose, OPGs display structural diversity through the linkage of β-glycosidic bonds, and can be decorated with succinyl and methylmalonyl substituents, responsible for the anionic properties of these polysaccharides [131]. The first protein to be found o be responsible for succinylation of OPG’s was MdoC in *E. coli* [5], identified through a screen for *E. coli* K-12 mutants lacking OPG succinylation. A similar approach was used to identify an OPG succinyltransferase in *Rhodobacter sphaeroides* named OpgC [134]. These stand-alone AT3 proteins contain homologues in many other Gram-negative bacteria, including homologues in the animal and human pathogen *Brucella abortus* and the plant pathogen *Dickeya dadantii* where both proteins succinylate their respective OPGs [135, 136].

The ECA is a conserved polysaccharide found across all enterobacteria [137], although its biological function still remains rather enigmatic [138]. ECA can be found in the outer membrane via linkage to phosphoglyceride [139], as a cyclic form in the periplasm [140], and, in bacterial strains incapable of producing O-antigens, it can be bound to lipid A [141]. *E. coli* K-12 ECA is composed of repeating units of three amino sugars, namely GlcNAc -α(1-4)-ManNAcA-β(1-4)-Fuc4NAc with an acetyl substituent on C6 of the GlcNAc residue [142]. The *wecH* (*yiaH*) gene encodes an AT3 protein that is essential for *O*-acetylation of this GlcNAc residue in *E. coli* [143]. This gene is not encoded within the main biosynthetic gene cluster for the ECA yet is present in many Enterobacteriaceae. WecH was also substituted in place of PatA, an MBOAT protein, and able to function with PatB to result in peptidoglycan *O*-acetylation; indicating that direct transfer of an acetyl group between an AT3-only protein and soluble SGNH domain (PatB) can occur [143] and also supporting the idea that SGNH domains could cross talk to both MBOAT and AT3 proteins in the membrane.

ECA has been suggested to play a role in *Salmonella* virulence, connected to resistance against bile salts [144] and has a range of other proposed biological functions [138], including a recent discovery of role for the cyclic periplasmic form in maintaining the outer membrane permeability barrier [145]. However, a clear role for the acetylation of ECA has yet to be presented.

### Secreted glycosylated products – Nod factors and antibiotics

The penultimate class of molecules that are targets for the action of AT3 proteins are secreted chemicals that contains sugars, either as the main part of their structure, such as in the Nod factors, or due to part of a more complex structure being modified by a sugar and then later tailored via acylation, as in the examples of the macrolide antibiotics.

The Nod factors are lipo-chitooligosaccharide molecules produced by bacteria that are able to initiate root nodule development during the establishment of nitrogen fixing symbiotic relationship with legumes [146]. They generally consist of a β-1,4-linked N-acetyl-d-glucosamine backbone with a lipid moiety on the non-reducing end and various other substitutions on the monosaccharides at either end of the chain. These structural modifications are orchestrated by expression of different biosynthesis genes (*nod* genes) and can affect the biological activity of Nod factors with different plant hosts [147, 148]. The AT3 protein encoded by the *nodX* gene was first identified as being essential for *Rhizobium leguminosarum* biovar *viciae* strain TOM to nodulate Afghanistan peas [9] (Fig. 2), which has been suggested to be through the *O*-acetylation of C6 of the reducing GlcNAc residue of the *R. leguminosarum* biovar *viciae* nodulation factor Nod*RIv*-V [149]. This modification has been speculated to have some role in Nod factor perception through interaction with the plant *sym2* gene product, whose activity depends on the structure of Nod factors secreted by the infecting bacterium [146, 150]. Similarly, NolL is an AT3 protein identified from *Rhizobium loti* (* Mesorhizobium loti *) that functions as an *O*-acetyltransferase, transferring an acetyl group specifically to the fucose residue of a fucose lipo-chitooligosaccharide [151]. Acetyltransferase activity of NolL has been associated with increased Nod factor production involved in root nodulation [151].

The macrolide antibiotics are natural products that contain a macrocyclic lactone ring that can be decorated with deoxy-sugars [152] and which act through inhibition of protein synthesis [153]. Various mechanisms of resistance to these compounds have been observed in bacteria, such as modification of the target and the drug itself, and therefore identifying mechanisms to introduce variation in these compounds is of high clinical importance [10]. In the macrolide carbomycin, one of the rare sugars added to the macrolide ring is mycarose [154, 155]. In the related antibiotic spiramycin this is unmodified, but in carbomycin the mycarose is acylated with an isovaleryl group, which requires the action of the AT3 protein CarE from *Streptomyces thermotolerans* [155]. This is interesting as this is a longer chain acyl modification than the frequently found acetyl- or succinyl-transferase activities of AT3 proteins (Fig. 2). Also, unlike most of the AT3 proteins there is evidence of substrate promiscuity in CarE; Arisawa *et al.,* [156] demonstrated that CarE (known as AcyB1 in their study) expressed in *Streptomyces lividans* was able to produce 4-O-isovaleryl modifications of the related macrolide tylosin, as well as 4-*O*-acetyl, 4-*O*-propionyl and 4-*O*-butyryltylosin modifications. In similar experiments MdmB was identified from *Streptomyces mycarofaciens* as a 3-*O*-acyltransferase that catalyses the addition of acetyl and propionyl groups to position 3 of the lactone ring in 16-member macrolide antibiotics like midecamycin and spiramycin [8]. Subsequently, further macrolide antibiotic acyltransferases AcyA, Mpt and MidE have been discovered in species of the genus *Streptomyces* and these genes have been used to manipulate engineered strains that produce modifications to the structures of common macrolide antibiotics [157, 158].

In addition to antibiotics, there are further examples of microbial natural products that are modified by AT3-only proteins. Chromomycin A_3_ and Ansamitocins are bacterial compounds that, amongst other roles, have been found to display antitumour activity.

Chromomycin A_3_ is an aureolic acid type compound from *Streptomyces griseus* which is also used as a fluorescent DNA stain and antimicrobial agent [159]. The AT3 only protein CmmA was first identified as a gene within the biosynthetic cluster for chromomycin A_3_ and was then functionally characterised by Menéndez *et al.* [160] to be responsible for the *O*-acetylation of C-4 of the rare sugars l‐chromose B and d‐oliose that form part of the structure of chromomycin A_3_. The acetylation modifications are functionally important as the parental compound lacking acetyl groups had reduced antibiotic and antitumour activity. Menéndez *et al.* [160] were also able to show that this modification takes place after the sugar residues have been added to the Chromomycin A3 precursor. García *et al.* [161] showed that CmmA exhibits both acyl donor and acceptor substrate flexibility as it is able to produce differently acetylated derivatives of exogenously added mithramycin, another structurally related antitumour compound and is also able to use acetyl-, isobutyryl‐ and propionyl‐CoA to perform these modifications (Fig. 2).

Ansamitocins are antimicrobials that also display antitumour activity and are synthesised by the Actinobacterium *Actinosynnema pretiosum* [162]. The AT3 protein Asm19 has been proposed to be the 3-*O*-acyltransferase responsible for catalysing the attachment of the acyl side chain of the ansamitocins, which are essential for bioactivity [163]. This was supported by Moss *et al.,* [164] who found that Asm19 had high substrate acceptor specificity paired with donor substrate flexibility as it was able to use acetyl-, propionyl-, isobutyryl-, butyryl-, or isovaleryl-CoA as a substrate but could only add these groups to C3 of *N*-desmethyl-4,5-desepoxymaytansinol (an ansamitocin precursor) (Fig. 2). This indicates promiscuity in the donor substrate, but a highly specific acceptor substrate. This example is also interesting as this appears to be an example where the *O*-acylation is not occurring on a monosaccharide group of some kind, here occurring within a cyclic polyketide structure.

### Modification of glycosylated proteins in pili

The final class of AT3 targets is a single important protein in species of the genus *Neisseria *, that is glycosylated after synthesis and then further modified through *O*-acylation. This protein is the pilin protein component of a bacterial pilus, afilamentous structure often involved in surface adhesion and biofilm formation [165, 166]. Pilin proteins can be post-translationally modified by addition of glycans [167], which may have role in pathogenicity. Warren *et al.* [168], determined that PglI was responsible for acetylation of the tetrasaccharide structure which is *O*-linked to Ser63 of Pilin protein PilE in *Neisseria meningitidis* strain C311#3. C311 strains of *N. meningitidis,* possess a covalently linked glycan structure composed of Gal(β1–4),Gal(α1–3),[2,4-diacetamido-2,4,6-trideoxyhexose] (DATDH) [169]. Only the DATDH of this tetrasaccharide repeat is acetylated and Warren *et al.*, concluded that PglI is the acetyltransferase responsible for DATDH acetylation of the pilin tetrasaccharide. This was also supported by the experiments of Aas *et al.,* and Anonsen *et al.,* [170, 171] for *N. gonorrhoeae *. PglI has an AT3-SGNH fused domain architecture (Fig. 2). Although a specific function for the *O*-acetylation is not known, there is evidence that this somehow controls oligosaccharide chain length [171].

### Insight into AT3 function and mechanism from comparisons of diverse characterised systems

This first, to our knowledge, compilation of AT3 catalysed functions has brought together diverse literature from the bacterial domain where AT3 proteins decorate a plethora of sugar-containing structures. While the total spectrum of *O*-acylated glycans is even broader due to the presence of other cytoplasmic or MBOAT-catalysed reactions, the AT3 proteins have evolved to function in an extremely broad range of cellular processes and influence the virulence of many pathogens through alterations of the cell surface. Also, from this group of functionally characterized bacterial AT3 proteins, we have seen that the biotechnologically relevant structure xanthan and those with biotechnological potential (Nod factors), other EPS that contribute to virulence and also antibiotics are modified by AT3 proteins, providing a strong incentive to elucidate the basic principles of how these proteins work, both to explore targeting the process as new therapeutic, as well as for maximizing their activity to apply in biotechnological processes. Whether the substrate range of any specific AT3 protein can be artificially expanded, which could also be of benefit for applications, is an outstanding question.

One mechanistic element that has been drawn out by this large literature analysis relates to the location of the *O*-acylation reaction. From many of the most well characterised systems it is apparent that the site of acylation is extracytoplasmic. This would be consistent with the trouble the cells go to by using a membrane protein to couple transport of an intracellular acyl-group with acylation of an extracytoplasmic sugar. The presence of disulfide bonds in structures of fused SGNH domains that are required for the acyltransferase function is only consistent with them being located in anextracytoplasmic location [11]. Given these data, it is worth considering two systems where the current model of biosynthesis of the acylated glycans is proposed to occur in the cytoplasm, namely the Neisseria PglI system [171] and the *Corynebacterium glutamicum* TmaT systems [80]. PglI is an AT3–SGNH protein with a typical SGNH domain, indicating a periplasmic site of action, while TmaT is a stand-alone AT3 protein. In both cases many uncertainties remain in the models for the overall biosynthesis of either the glycosylated pilin subunit for PglI or the mycolic acid for TmaT and hence the position and subcellular location of the *O*-acetylation stages, and only further detailed analysis will allow resolution of the apparent discrepancies with other family members. The structure or a deeper understanding of the mechanism of action of AT3 family proteins could resolve these differences.

The second piece of mechanistic insight is provided by a number of the named systems where *in vitro* studies have demonstrated that acyl-CoA can function as the acyl donor for an AT3 reaction. A nice example comes from the CmmA protein involved in macrolide *O*-acetylation. Using purified and washed membrane fractions containing CmmA, Garcia *et al.* [161], were able to demonstrate that acetyl-CoA was sufficient for the resulting modification of the antibiotic, providing strong support for the hypothesis that acyl-CoA molecules are the physiological acyl-donors for AT3 proteins, rather than this being mediated by other candidates like the acyl-carrier protein (ACP). Similar data has been presented for TmaT [80] and most recently a clear identification of acetyl-CoA as the substrate for OatA has been presented [30]. In this study the authors also showed that acetyl-CoA cannot be used as the donor for the SGNH domain of OatA, but is used by the AT3 domain and subsequent transfer of the acetyl group to a muropeptide acceptor requires the SGNH domain to be catalytically active, demonstrating the involvement of both domains to transfer the acetyl group from acetyl-CoA to its acceptor. As in this system, like the OafA system for LPS *O*-aceylation [11], there is evidence for the concerted actions of both domains, it now makes us want to understand how stand-alone AT3 proteins can function. Clearly much more about the mechanism of these proteins needs to be elucidated to first understand their function and secondly think about how to inhibit these enzymes. However, there is one common element to all the types of AT3 proteins described in that they must all bind the acyl-donor molecule in a consistent manner and this is reflected by the discover of essential residues that are widely conserved in AT3 proteins that are likely to be involved in this process [11, 30, 32]. Essential residues, RxxR and R/K-X10-H, are predicted to be able to bind the acyl-CoA molecule, potentially allowing it to penetrate deep into the membrane and the discovery of a potential catalytic tyrosine in OatA in the AT3 domain indicates a position in which the initial acyl-transfer might occur [30]. Beyond this it is unclear how this is then moved to the SGNH domain and then onto the acceptor molecule and how this occurs in stand-alone AT3 systems, in which presumably the AT3 domain can recognise the acceptor directly and catalyse the acetyltransferase reaction.

While the function of fused SGNH domains is now becoming clearer through work on OafA, Oac and OatA, we also note that there is another protein architecture of AT3 proteins where they have a C-terminal fusion to an alanine racemase domain instead of an SGNH domain. This is exemplified by a protein called VanT from *Enterococcus casseliflavus* ATCC 25788 [172], where the gene has been experimentally demonstrated to be required for vancomycin resistance. The C-terminal domain is thought to catalyse the conversion of l-serine to d-serine, while the role of the AT3 domain is unknown, although has been proposed to be an l-serine transporter [173]. We note here that fusions of AT3 proteins to C-terminally located ‘alanine racemases’ are the second most common type of fusion seen for AT3 domains (see Pfam architectures for PF01757), and would argue that they deserve some experimental analysis to determine the function of the AT3 domain and its partner protein.

Finally, a structure of an AT3 protein is also still awaiting solution and would, naturally, provide great insight into the mechanism of action of these important membrane-bound enzymes. While an MBOAT structure is known [25], the results of an analysis of sequence conservation of AT3 proteins completed by Pearson *et al.* [11], compared with that of the known DltB MBOAT structure, indicate that these proteins appear to be unrelated and we suggest that they have different folds. Also a recently determined topology model of OatA has added to our knowledge of the structure of AT3s [30], but given the highly atypical structure of MBOAT the fusion technique used in that study certainly needs further support from structural biology approaches, which is still a pressing challenge for the community.

In conclusion, in this review we have highlighted the diverse range of acylations that AT3 proteins can be involved in, discussed the different architectures of the proteins and touched on the limited but exciting work trying to tease apart the mechanisms of these important membrane-bound enzymes.
